# Increased expression of circulating miRNA-93 in women with polycystic ovary syndrome may represent a novel, non-invasive biomarker for diagnosis

**DOI:** 10.1038/srep16890

**Published:** 2015-11-19

**Authors:** T. Sathyapalan, R. David, N. J. Gooderham, S. L. Atkin

**Affiliations:** 1 Academic Diabetes, Endocrinology and Metabolism, Hull York Medical School, University of Hull, UK; 2 Faculty of Medicine, Department of Surgery & Cancer, Imperial College, London, UK; 3 Weill Cornell Medical College Qatar, Education City PO Box 24144, Doha, Qatar

## Abstract

MicroRNAs (miRNA) are a novel class of small noncoding single-stranded RNA molecules that regulate gene expression. There is increasing evidence of their importance in polycystic ovary syndrome (PCOS). The objective was to determine if miRNA-93 and miRNA-223 are differentially expressed in the circulation of women with PCOS compared to age matched women. A case–control study comparing women with PCOS (n = 25) to age and weight matched controls (n = 24) without PCOS was performed. MiRNA-93 and miRNA-223 were determined by total RNA reverse transcription. Both miRNA-93 and miRNA-223 were significantly increased relative to the control group (p < 0.01, p = 0.029 respectively). In both groups there was no correlation of either miRNA-93 or miRNA-223 with insulin, HOMA-IR, HOMA-β or testosterone levels. The area under the receiver operator characteristic curve for miR-223 and miR-93 was 0.66 and 0.72 respectively, suggesting miR-93 is a more efficient biomarker than miR-223 for diagnosis of PCOS. The combination of the two miRNAs together, tested using multiple logistic regression analysis, did not improve the diagnostic potential. In conclusion, circulating miRNA-93 and miRNA-223 were higher in women with PCOS compared to age and weight matched controls independent of insulin resistance and testosterone levels, and miR-93 may represent a novel diagnostic biomarker for PCOS.

Polycystic ovary syndrome (PCOS) is one of the most common endocrine disorders and affects 6–20% of reproductive-aged women^1^,^2^,^3^. Seventy five to ninety per cent of PCOS patients demonstrate insulin resistance (IR) above and beyond that predicted by body mass, race, or age^4^,^5^, resulting in compensatory hyperinsulinemia^6^ and an increased risk for type 2 diabetes mellitus (T2DM)^7^ and cardiovascular disease^8^. Cellular mechanisms leading to IR in PCOS remain unclear although a post-binding defect in receptor signalling has been suggested^9^. However, tissue changes in the adipocyte function, including the stimulation of glucose transport^10^ and GLUT4 production^11^, have been described in women with PCOS^12^,^13^,^14^.

MicroRNAs (miRNAs) are a novel class of small noncoding single-stranded RNA molecules 18–24 nucleotides long that regulate gene expression at the posttranscriptional level. Evolutionarily conserved, miRNAs bind to the 3′ un-translated regions of messenger RNAs (mRNAs), and induce degradation or inhibition of protein translation. MiRNAs possess many critical regulatory functions in a wide range of biological processes such as cell proliferation, differentiation, survival and apoptosis, and the stress response^15^. Any one particular miRNA has the potential to modulate the expression and functions of hundreds of downstream target genes^16^. In addition, the existence of feedback regulation mechanisms between miRNA, their targets, and their products allows for amplification or inhibition of a specific signal. Hence, alteration of even a handful of miRNAs can possibly result in dramatic deregulation of physiologic cellular functions. Emerging evidence suggests an increasing role for miRNA in both type 1 and type 2 diabetes with the potential for their use as novel disease biomarkers^17^. Tissue changes, follicular fluid alterations and circulating miRNA have been described in PCOS^18^,^19^,^20^,^21^ with the suggestion that the expression of three miRNAs were elevated compared to controls that may act as novel biomarkers^18^. However, their expression is likely to be complex with evidence that miRNA-21, miRNA-27b, miRNA-103, and miRNA-155 may be differentially expressed in obesity and in PCOS^21^. Recently a study identified the down regulation of the insulin sensitive-glucose transporter GLUT4 gene expression by miRNA-93 in adipose tissue^19^, with the suggestion that miRNA-223 may have an undefined role in insulin resistance in PCOS. A subsequent study showed that miRNA-93 in adipose tissue was overexpressed in PCOS patients with insulin resistance though discordant for expression of the host gene MCM7^22^. In view of these findings this study was performed to determine if miRNA-93 and miRNA-223 were found in the circulation and to determine their correlation to the metabolic indices found in PCOS compared to weight matched normal controls.

## Materials and Methods

Twenty-five medication naïve women with PCOS and biochemical hyperandrogenaemia (age 18–45 years) who presented sequentially to the department of endocrinology and who fulfilled the criteria of the study were recruited from the local PCOS biobank (ISRCTN70196169). Twenty-five normal women (age 20–44 years) were recruited from the PCOS biobank and were age and body mass index (BMI) matched to the PCOS subjects for inclusion into this study. All of the control women had regular periods, no clinical or biochemical hyperandrogenemia, no significant background medical history and none of them were on any medications including oral contraceptive pills or over the counter medications. All women with PCOS and control women were Caucasian. Subject demographics are shown in Table 1.

The diagnosis of PCOS was based on all three diagnostic criteria of the Rotterdam consensus, namely clinical and biochemical evidence of hyperandrogenemia (Ferriman-Gallwey score >8; free androgen index >4 respectively), oligomenorrhea or amenorrhea and polycystic ovaries on transvaginal ultrasound^23^.Liver ultrasound was performed at the same time to exclude non alcoholic fatty liver disease. Study participants had no concurrent illness, were not on any medication for the preceding nine months including oral contraceptive pills or over the counter medications and were all non-smokers. None of the patients had successful pregnancy or miscarriage at least five year prior to the study entry. Diabetes was excluded by a 75 g oral glucose tolerance test. Non-classical 21-hydroxylase deficiency, hyperprolactinaemia, Cushing’s disease and androgen-secreting tumours were excluded by appropriate tests. All patients gave informed consent. This study was approved by the Newcastle & North Tyneside Ethics committee. Methods were carried out in accordance with the approved guidelines.

Blood samples were taken after an overnight fast and serum was stored frozen at −80 ^°^C pending analysis. Serum testosterone and androstenedione were measured by isotope dilution liquid chromatography- tandem mass spectrometry (Waters Corporation, Manchester, UK). Sex hormone binding globulin (SHBG) by an immunometric assay with fluorescence detection on the DPC Immulite 2000 analyzer using the manufacturer’s recommended protocol. The free androgen index was obtained as the total testosterone x100/SHBG. Serum insulin was assayed using a competitive chemiluminescent immunoassay performed on the manufacturer’s DPC Immulite 2000 analyzer (Euro/DPC, Llanberis, UK). The analytical sensitivity of the insulin assay was 2 μU/ml, the coefficient of variation was 6%, and there was no stated cross-reactivity with proinsulin. Plasma glucose was measured using a Synchron LX 20 analyzer (Beckman-Coulter), using the manufacturer’s recommended protocol. The coefficient of variation for the assay was 1.2% at a mean glucose value of 5.3 mmol/liter during the study period. The insulin resistance was calculated using the HOMA method [HOMA-IR=(insulin x glucose)/22.5], and pancreatic beta cell sensitivity measured by HOMA-β [HOMA-β = (20 × insulin)/glucose −3.5]. Serum CRP was measured by the high-sensitivity method on a Beckman DXC analyzer.

### RNA extraction

Total RNA was extracted from plasma from 25 women with PCOS or 25 healthy women matched for age and BMI using the MiRVana PARIS kit (Life Technologies, Paisley, UK) following manufacturer’s instructions with the following modifications. Prior to addition of the acid-phenol:chloroform, 150Amoles of c-elegans miRNA-39 (Ambion, Life Technologies) spike-in was added to the sample as an RNA carrier. Total RNA was eluted by adding 10 μl of RNAse-free water to the membrane of the spin column and incubating for 2 min before centrifugation at 10000xg for 30 s at room temperature, which was repeated with a further 10 μl. The RNA was stored at −80 °C.

### Reverse transcription and qPCR

Total RNA was reverse transcribed using the TaqMan MicroRNA Reverse Transcription Kit (Life Technologies, Paisley, UK) following manufacturer’s instructions. Mature miRNA TaqMan assays were purchased from Life Technologies and the generated cDNA amplified using the Taqman 2x Universal PCR master mix, No AmpErase UNG (Life Technologies), with each reaction performed in triplicate. The amplification was performed in a StepOnePlus PCR System (Life Technologies) in 96 well plates. The amplification curves were analysed using the StepOne software (Life Technologies) and the comparative Ct Method (ΔCT Method)^24^. Calibration was based on the expression of the c-elegans miR-39 spike-in.

### Statistical analysis

For a significant change in miRNA measurements power was based on a 50% increase or decrease of the relative change to control, with a common standard deviation of 0.5^19^. A sample size of 23 participants per group to has 90% power, 1% significance (two-tailed) was calculated (n-query software, USA).

An unpaired t test was used to compare changes from baseline for the biochemical data and clinical observations within groups. The Wilcoxon signed rank test was applied to biochemical data that violated the assumptions of normality when tested using the Kolmogorov-Smirnov test. For all analysis, a two-tailed *P* ≤ 0.05 was considered to indicate statistical significance. Statistical analysis was performed using SPSS for Windows NT, version 22.0 (SPSS Inc., Chicago, IL). Data are reported as mean (SEM).

A one-way ANOVA with post-hoc Dunnett’s test performed (GraphPad Software, La Jolla California USA) was used to test for statistical significance between miRNA expression in plasma from patients with PCOS compared to the control group. A ROC curve was plotted for each miRNA individually, and in combination, to determine their discriminating effects. The sensitivity and specificity of detecting cases and controls were assessed by the area under the ROC curve (AUC) and 95% confidence interval (CI). Multiple logistic regression and ROC curve analysis was performed in R^25^.

*In silico* analysis was carried out using miRWalk, which predicts targets based on the mRNA 3' UTR region produced by the miRWalk program and also presents results from other programs with target gene prediction, of which miRanda, TargetScan and DIANA were used. Only targets predicted by all 4 databases were used for pathway analysis by Ingenuity Pathway Analysis (IPA; Qiagen, Redwood City).

## Results

Both groups were well matched for age, weight and BMI (Table 1). Of the 25 PCOS patients 8 had a BMI of 25 kg/m^2^ or less and of those only 1 of 8 had biochemical hyperandrogenemia with an elevated FAI, whilst of those 17 PCOS patients with a BMI greater than 25 kg/m^2^, only 3 of 17 did not have a raised FAI. In the PCOS group insulin was significantly increased (p = 0.03), HOMA-IR did not differ (p = 0.07), there was a significant increase in HOMA-β (p = 0.001) and testosterone was significantly elevated (p = 0.03) (Table 1).

**Figure 1:**
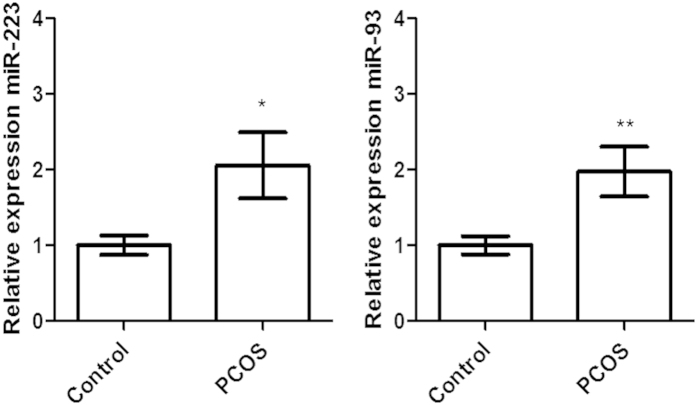
Increased expression of microRNA(miR)-223 and miR-93 in plasma from patients with PCOS (n = 25) relative to that expressed in the control age and BMI matched population (n = 24). Data are means ± SEM, significance compared to control (one-way ANOVA with Dunnett’s post-test; *P ≤ 0.05, **P ≤ 0.01).

The expression of both miRNA-93 and miRNA-223 was significantly increased in PCOS patients relative to the control group (p = 0.009 and p = 0.029, respectively) (Fig. 1). In the control group there was no correlation of either miRNA-93 or miRNA-223 with any of the metabolic and hormonal parameters including insulin (0.4;p = 0.09, 0.2;p = 0.5 respectively), HOMA-IR (0.4;p = 0.07, 0.2;p = 0.3 respectively), HOMA- β (0.1;p = 0.8, 0.2;p = 0.3 respectively) or testosterone (0.04;p = 0.9, 0.1;p = 0.7 respectively). In the PCOS group there was no correlation of either miRNA-93 or miRNA-223 with any of the parameters including insulin (0.2;p = 0.4, 0.1;p = 0.5 respectively), HOMA-IR (0.2;p = 0.4, 0.1;p = 0.7 respectively), HOMA- β (0.07;p = 0.8, 0.2;p = 0.3 respectively) or testosterone (0.3;p = 0.1, 0.1;p = 0.6 respectively).

**Figure 2:**
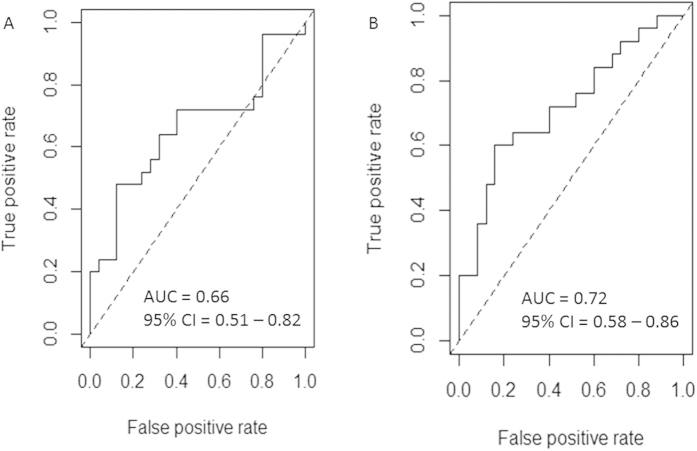
ROC curve analysis of (A) miR-223 and (B) miR-93 to discriminate women with PCOS from healthy controls.

To evaluate the diagnostic value of miR-223 and miR-93, ROC curves were plotted and the AUC calculated (Fig. 2). The sensitivity and specificity for miR-223 and miR-93 were calculated at the cut-off values of 1.2 and 1.1 respectively, at which the largest sensitivity and specificity was defined as the optimal diagnostic point. The sensitivity, specificity and AUC of miR-223 and miR-93 were 0.56, 0.72 and 0.60 (95% CI: 0.51–0.82), and 0.64, 0.76 and 0.72 (95% CI: 0.58–0.86) respectively. The results show that miR-93 is more efficient than miR-223 as a biomarker for the diagnosis of PCOS. Multiple logistic regression and ROC curve analysis was used to determine whether the combination of miR-93 with miR-223 had improved diagnostic potential over miR-93 alone. The results showed that the combination of the two miRNAs did not improve beyond the diagnostic value of miR-93 alone (sensitivity, specificity and AUC of miR-223 and miR-93 combined were 0.64, 0.76 and 0.73 (95% CI: 0.59–0.87) respectively).

Following target gene prediction for miR-223 and miR-93 using miRWalk, miRanda, TargetScan and DIANA, a list of possible target genes identified by all 4 programs was obtained and analysed using IPA to generate a list of the top canonical pathways likely to be specifically controlled by these miRNAs. As shown in Table 2 and Table 3, genes that are predicted to be targeted by these miRNAs are involved in pathways including cancer, stem cell pluripotency and hepatic growth factor (HGF) signalling.

## Discussion

This is the first study to show the elevated plasma levels of both miRNA-93 and miRNA-223 in women with PCOS particularly when weight was accounted for. These increased miRNA levels were independent of insulin, insulin resistance and of hyperandrogenaemia in women with PCOS.

It may have been anticipated that a strong correlation to insulin resistance would be found given the recent report of miRNA-93 and miRNA-223 up-regulation in PCOS adipocytes, and the *in vitro* demonstration of down-regulation of GLUT4 gene expression^19^. In a subsequent study miRNA-93 in adipose tissue correlated with PCOS and insulin resistance though its host gene MCM7 was discordantly downregulated^22^. However, the PCOS and control women were not matched for BMI or insulin resistance, which may explain the discrepancies between that study and ours. Other reasons for this apparent lack of correlation are likely to be multifactorial as it is unclear how circulating levels of miRNA may relate to tissue function and effect. However, the top canonical pathways identified peroxisome proliferator receptor (PPAR), insulin like growth factor- 1 (IGF-1) and angiopoietin signaling as being potentially regulated by miR-223, all of which have been shown to play a role in PCOS. PPAR signaling has been shown to be important in insulin resistance, hyperandrogenism, endometrial response and in the ovary in PCOS,^26^,^27^ with PPAR gamma stimulation by pioglitazone being proposed as a potential therapeutic modality^26^. IGF-1 is a potent mitogenic factor and it appears necessary for ovarian follicle development^28^, and has been shown to be involved in pre-antral follicle growth in PCOS^29^. Angiopoietin 1 and 2 are essential for ovarian function and are preferentially increased in ovarian stimulation in PCOS^30^ . The canonical pathways identified both Nerve Growth Factor (NGF) Signalling and Hepatic Growth Factor (HGF) Signalling in the top 5 canonical pathways for potential regulation by miR-93. NGF may have an important role in the ovary with excessive production being associated with cystic ovarian morphology in rodent models akin to that found in women with PCOS^31^,^32^. HGF is an adipokine that is also found in the ovary^33^ but has been associated with obesity and metabolic syndrome^34^,^35^,^36^ with the suggestion that it has importance in insulin resistance associated compensatory mechanisms leading to an increase in insulin secretion through enhanced beta cell mass^35^. Furthermore, of those identified, molecular mechanisms of cancer was common for both miRNAs. The current study indicates that analysis of miRNAs offers potential insights into pathways that are affected in PCOS. The data, although limited to 2 miRNAs in the present study, warrant further in depth investigation into the role of miRNA in PCOS. One approach could be to examine the circulating miRNAome in such patients.

The canonical pathways in this study identified different targets for miR-223 and miR-93 to previous studies, but this is not surprising given that miRNAs differ in their tissue specific actions that are likely to be tissue and circumstance specific. Examples include miR-223 overexpression in cardiomyocytes increased GLUT4 expression^37^, reduced expression of miR-223 in breast cancer cell affects mediators of invasion^38^, and miRNA-93 and miRNA-223 have been associated with dysregulation in diabetes^17^.

Circulating miRNAs are very stable to enzymatic degradation and physical conditions such as freeze/thaw, and can be stored for months without their degradation^39^. As such they are attractive as biomarkers for disease diagnosis and monitoring. Therefore specific miRNA or combination of miRNAs may become a reliable biomarker for the diagnosis of PCOS, which may help distinguish the differing phenotypes identified by the Rotterdam criteria. Whilst the sensitivity and the specificity shown in the receiver operator curves are too low as a single diagnostic test, miRNA-93 may be a biomarker to support a positive diagnosis of PCOS rather than the diagnosis being one of exclusion that is currently the case, which would be of value in adolescence or in the perimenopausal/menopausal woman where diagnosis is difficult^40^. Our study demonstrates that plasma levels of miR-93 can distinguish patients with PCOS from healthy controls with more sensitivity and specificity than miR-223, and as such, circulating miR-93 may represent a biomarker for PCOS.

In a recent study, miR-222, miR-146a and miR-30c were found to be significantly increased in PCOS patients with the combination of the three miRNAs showing potential promise for the more efficient diagnosis of PCOS^18^. In the current study, however, the combination of miR-93 with miR-223 did not improve the diagnostic potential of miR-93 alone. The scope of miRNA involved in the pathophysiology of PCOS is unknown and likely extensive; in a rat PCOS model 24% of 349 miRNAs were differentially expressed compared to control following dihydrotestosterone treatment^41^.

The strengths of the current study included age and weight matching of the PCOS with normal subjects that circumvented miRNA expression associated with weight differences in PCOS^21^. Other strengths were the inclusion of all 3 criteria for PCOS diagnosis, the study was powered to detect a difference in miRNA, and liver ultrasound and alanine transferase measurements excluded non-alcoholic fatty liver disease that is associated with miRNA expression^42^. There were no changes in markers of inflammation, hsCRP or white cell count in this study, that may be elevated in PCOS when obesity has not been accounted for, which is important as both miRNA-93 and miRNA-223 have been associated with inflammation^43^,^44^.

This study is limited by the small cohort that was employed and a larger study specifically powered for each or the phenotypes that are described in the Rotterdam diagnostic criteria is needed for validation, and comparison with the NIH and Androgen Excess PCOS Society guidelines^40^. This study included women who satisfied all of the three Rotterdam diagnostic criteria for rigorous phenotyping; therefore, the expression of these miRNA may only define the most severe metabolic phenotype and may not be generalizable to those PCOS women diagnosed from only two out of three criteria and further studies are necessary to clarify this. Caution needs to be expressed in extrapolating the results of insulin resistance determined by the HOMA method reported here to measures of insulin resistance by other methods, such as insulin clamps or intravenous glucose tolerance tests. However, there is evidence to show that there is strong correlation between HOMA and other methods^45^,^46^,^47^,^48^.

In summary, PCOS subjects with elevated insulin and testosterone levels, showed higher circulating levels of miR-93 and miR-223 compared to age and weight matched controls, though the elevation in the miRNA levels was not related to either insulin resistance or hyperandrogenaemia. Moreover, miR-93 elevation in the plasma may represent a novel, non-invasive biomarker for the diagnosis of PCOS.

## Additional Information

**How to cite this article**: Sathyapalan, T. *et al.* Increased expression of circulating miRNA-93 in women with polycystic ovary syndrome may represent a novel, non-invasive biomarker for diagnosis. *Sci. Rep.* **5**, 16890; doi: 10.1038/srep16890 (2015).

## Figures and Tables

**Table 1:** Demographics, biochemical and clinical markers for the PCOS and control group (unpaired t test)

	Normal n = 25	PCOS n = 25	P value
	Mean (SD)	Mean (SD)	
Age (years)	32.2(7.7)	32.1(9.0)	0.97
Weight (kg)	76.0(18.8)	77.4(16.3)	0.79
BMI (kg/m^2^)	27.1(5.8)	28.8(5.4)	0.31
Fasting glucose (mmol/l)	4.7(0.4)	4.8(0.6)	0.56
2 Hour glucose (mmol/l)	4.9(1.2)	5.7(1.3)	0.06
Androstenedione (nmol/l)	8.4(5.1)	10.7(6.7)	0.25
ALT (IU/L)	24.3(14.4)	23.3(12.7)	0.79
Insulin (μU/ml)	6.8(3.7)	10.2(6.4)	0.03
HOMA-IR	1.5(0.9)	2.3(1.7)	0.07
HOMA-β	38.4(66.1)	158.8(89.7)	0.001
Testosterone (nmol/L)	1.2(0.7)	2.3(1.6)	0.03
SHBG (mmol/L)	81.8(105.8)	52.6(53.1)	0.29
FAI	2.6(1.7)	11.1(18.9	0.13
hsCRP (mg/l)	2.3(3.8)	2.8(3.9)	0.65
miR-93 expression (relative to control group)	1.0(0.6)	2.0(1.6)	0.009
miR-223 expression (relative to control group)	1.0(0.7)	2.01(2.2)	0.029

(BMI, body mass index; FAI, free androgen index; SHBG, sex hormone binding globulin; HOMA-IR, homeostatic model assessment-insulin resistance; HOMA-β, homeostatic model assessment-insulin beta cell sensitivity; hsCRP, high sensitivity C-reactive protein.).

**Table 2:** Top canonical pathways predicted by *in silico* analysis using 3′ UTR target prediction software to be regulated by microRNA-223.

Name	p-value
Top Canonical Pathways	
PPAR Signalling	6.24E-05
Prostate Cancer Signalling	1.57E-04
IGF-1 Signalling	1.71E-04
Angiopoietin Signalling	1.76E-04
Molecular Mechanisms of Cancer	3.69E-04

**Table 3:** Top canonical pathways predicted by *in silico* analysis using 3′UTR target prediction software to be regulated by microRNA-93

Name	p-value
Top Canonical Pathways	
Molecular Mechanisms of Cancer	2.38E-06
Nerve Growth Factor Signalling	3.53E-06
Human Embryonic Stem Cell Pluripotency	4.62E-05
Hepatic Growth Factor Signalling	5.76E-05
Pancreatic Adenocarcinoma Signalling	8.21E-05
